# Serum fibroblast growth factor 19 is decreased in patients with overt hypothyroidism and subclinical hypothyroidism

**DOI:** 10.1097/MD.0000000000005001

**Published:** 2016-09-30

**Authors:** Yaxin Lai, Haoyu Wang, Xinghai Xia, Zhaojun Wang, Chenling Fan, Hong Wang, Hongmei Zhang, Shuangning Ding, Weiping Teng, Zhongyan Shan

**Affiliations:** The Liaoning Provincial Key Laboratory of Endocrine Disease, Department of Endocrinology and Metabolism, The First Affiliated Hospital of China Medical University, China Medical University, Shenyang, China.

**Keywords:** FGF19, hypothyroidism, thyrotropin

## Abstract

As a newly emerging metabolic regulator, accumulating evidence suggests that the circulating fibroblast growth factor 19 (FGF19) level correlated with lipid and glucose metabolism. Several independent groups have found that FGF19 was highly likely associated with multiple metabolic disorders. Thyroid dysfunction is believed to be associated with metabolism diseases. However, to date, few studies have investigated the role of FGF19 in patients with thyroid dysfunctions. For this purpose, a cross-sectional study was done to estimate the role of FGF19 in patients with different thyroid functions. Compared with the healthy control, the present study revealed that serum FGF19 levels were significantly decreased in overt hypothyroidism patients (78.7 [52.7–121.2] vs 292.4 [210.2–426.5] pg/mL, *P* <0.001). FGF19 concentration was also lower in the subclinical hypothyroidism group than it was in the healthy control group (95.8 [71.7–126.3] vs 292.4 [210.2–426.5] pg/mL, *P* <0.001). However, there was no significant difference in FGF19 level between the isolated thyroid autoantibody positive group and the healthy control group (252.0 [205.9–353.5] vs 292.4 [210.2–426.5] pg/mL, *P* >0.05). Also, serum thyroid stimulating hormone (TSH) was an independent predictor of FGF19. In conclusion, thyroid insufficiency but not thyroid autoimmunity may have impacted serum FGF19 concentrations. As the role of FGF19 is becoming more and more important in the pathogenesis of many metabolic diseases, we proposed that the thyroid hormone level should be taken into account when the serum concentration is explained. Further studies are needed to elucidate the role of FGF19 in the development of hypothyroidism.

## 1 Introduction

The fibroblast growth factor (FGF) family comprises more than 20 members with diverse functions, such as embryonic development, cell growth, and differentiation. By activating specific receptor, FGF15/19, FGF21, as well as FGF23, are endocrine factors involved in hormone-like metabolic effects. FGF19 is mainly synthesized in intestine and could be secreted into circulation.^[1,2]^ Previous studies, however, uncovered the effects of FGF19 on maintaining energy metabolism, presumably through its functions as an endocrine hormone. Indeed, compared with the wild-type mouse model, the transgenic mouse model with overexpressed FGF19 has lower levels of body weight and cholesterol. It suggests that serum FGF19 may play a vital role in regulating glucose metabolism and insulin sensitivity. Similar results were observed when high-fat-fed mice were treated with recombinant FGF19.^[3,4]^ Because of this novel significance in metabolic diseases, the research on FGF19 has attracted attention in academia.

Thyroid disorder is the second most common endocrine disease, which affects 3% to 13.5% of the population.^[5–7]^ TSH has long shown several regulatory functions of metabolic and energy homeostasis, including growth, body weight, thermogenesis, protein synthesis, and lipolysis.^[8]^ Our previous studies demonstrated that abnormal thyroid function is associated with an increased risk of metabolic disorders resulting in disruption of body homeostasis.^[9–12]^ Thyroid dysfunction is a risk factor of cardiovascular disease, nonalcoholic fatty liver disease (NAFLD), and a variety of metabolic diseases.^[9,10,13–16]^ However, to date, we know little about the molecular mechanism of how thyroid dysfunction accelerates the process of metabolic disorders.

Despite the previous findings of FGF19 and the well-established correlation of metabolic risks with thyroid dysfunction, few clinical studies have reported on the potential association of FGF19 with thyroid dysfunction. In general, autoimmune thyroiditis progresses through 3 clinical stages that are defined by thyroid function: the early stage (euthyroid with positive autoantibodies), the second stage (subclinical hypothyroidism), and the final stage (overt hypothyroidism). It has been found that these clinically defined disease stages are closely correlated to the severity of the disease. For this purpose, a cross-sectional survey was performed to estimate the relationship of FGF19 with thyroid dysfunction.

## 2 Materials and method

### 2.1 Subjects

**Figure 1:**
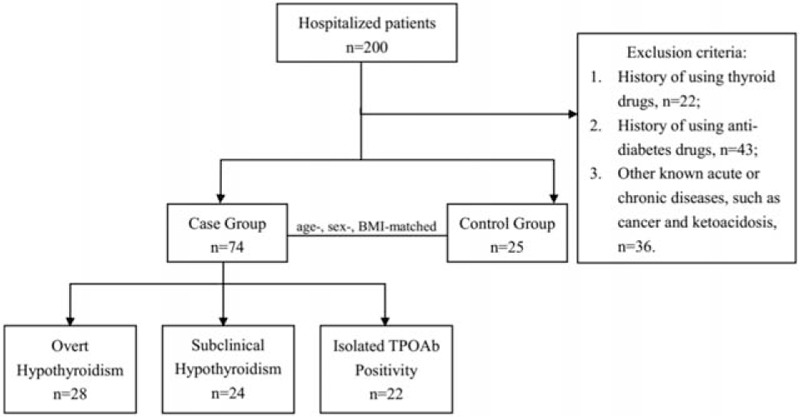
Flow graph of case recruitment.

To analyze the effect of thyroid function on FGF19 concentrations, 99 subjects were randomly recruited from patients attending the Department of Endocrinology and Metabolism at the First Hospital of China Medical University (Shenyang, China) from June 2012 to March 2014. In our study, age-, sex-, and body mass index (BMI)-matched subjects in the overt hypothyroidism group (OH; n = 28), subclinical hypothyroidism group (SCH; n = 24), isolated thyroid peroxidase antibody (TPOAb) positivity with euthyroid group (isolated TPOAb; n = 22), and healthy control group (HC; n = 25) were included and subjects who presented with any known acute or chronic illness, such as overt diabetes, necessitating treatment were excluded (Fig. 1).

This present study was approved by the Medical Ethics Committee of the First Affiliated Hospital of China Medical University and was conducted in accordance with approved guidelines and regulations.

### 2.2 Diagnostic criteria

Overt hypothyroidism (OH) was defined as a TSH>4.94 mIU/L and free thyroxine (FT4) <9.01 pmol/L, while subclinical hypothyroidism (SCH) as a TSH >4.94 mIU/L and FT4 within the normal range. Isolated TPOAb positivity (Isolate Ab) was defined as an isolated TPOAb concentration >300 IU/mL with normal TSH and FT4. And healthy control was defined as a subject with normal TSH, FT4, TPOAb, and TgAb.

### 2.3 Anthropometric and biochemical measurements

Height and weight were measured to the nearest 0.1 cm and 0.1 kg, respectively, and BMI was calculated as weight divided by height squared (kg/m^2^) at the time of blood sampling. A venous blood sample was taken after an overnight fasting. After clotting, blood specimens were separated by centrifugation for 15 minutes at 1000 g. Serum samples were subsequently stored in aliquots without preservative at −80°C for an average of 3 months until immediately before analysis of FGF19. Serum TSH, free triiodothyronine (FT3), FT4, TPOAb, and thyroglobulin antibody (TgAb) were tested with a super-sensitive chemiluminescence immunoassay (ARCHITECT system i2000SR, Abbott Laboratories, Chicago, USA), while alanine aminotransferase (ALT), aspartate aminotransferase (AST), gamma glutamyl transpeptidase (GGT), low-density lipoprotein (LDL), high-density lipoprotein (HDL), total cholesterol (TC), and triglyceride (TG) were measured using the Cobas Elesys 601 (Roche Diagnostics, Basel, Swiss)

### 2.4 Measurement of FGF19

FGF19 levels in the fasting serum were assessed using a sandwich enzyme-linked immunosorbent assay (ELISA; FGF19 Quantitative ELISA kit, R&D, Minneapolis). The procedure was in accordance with the manufacturer's instruction. The standard curve range for the assay was 15.6 to 1000 pg/mL. All samples were analyzed in duplicate. If duplicates had >15% CV, the sample was repeated.

### 2.5 Statistical analysis

Data are expressed as mean ± standard deviations for normally distributed variables, median with interquartile range for non-normally distributed variables, and as frequencies for categorical variables. In comparisons between the study groups, normally distributed variables were assessed using one-way analysis of variance (ANOVA) followed by Bonferroni correction in paired comparisons. Some variables failed the normality test; therefore, these variables were assessed using Kruskal–Wallis one-way ANOVA on ranks in groups, and pairwise comparisons were performed using Mann–Whitney rank sum test. Correlation analysis was performed using the Pearson correlation or Spearman rank correlation method. To identify independent relationships and adjust the effects of covariates, multiple linear regression analyses were performed including all parameters with highly significant correlations in the univariate analysis as covariates. Non-normally distributed variables were analyzed after log transformation was performed. All statistical analyses were performed with SPSS version 20.0 software.

## 3 Results

### 3.1 Circulating FGF19 levels were decreased in patients with hypothyroidism

**Figure 2:**
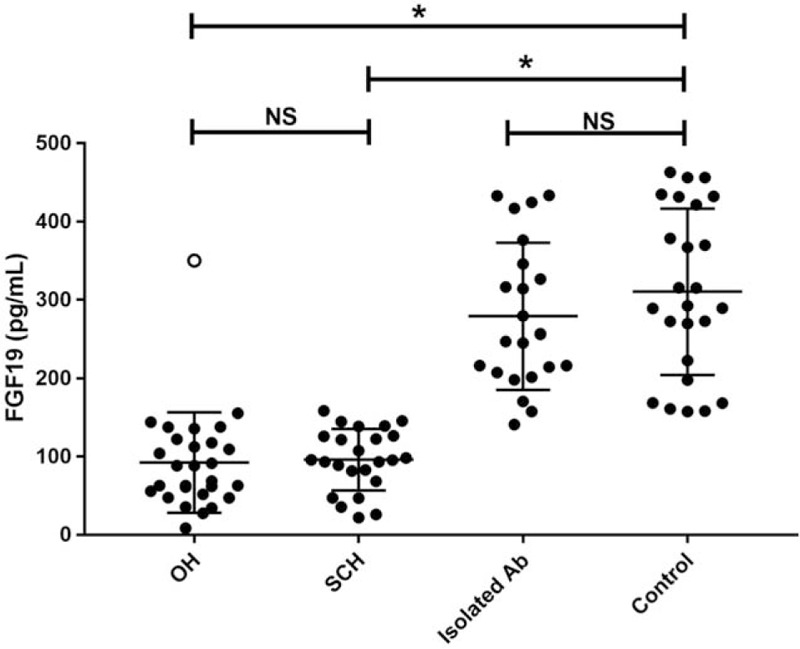
Circulating FGF19 level was decreased in patients with OH and SCH when compared with HC group and isolated Ab group. NS = not significant, OH = overt hypothyroidism, SCH = subclinical hypothyroidism; ^∗^*P* <0.05; ○, outlier.

Characteristics of patients with OH, SCH, isolated TPOAb positivity with euthyroid, and HC are described in Table 1. There were no significant differences in age, sex, BMI, SBP, DBP, and liver function between every 2 groups (all *P* values >0.05). However, LDL was higher in the OH group when compared with the HC group (*P* = 0.006), and LDL was also higher in the SCH group when compared with the HC group (*P* = 0.049). As shown in Fig. 2, serum FGF19 levels of patients with OH or SCH were significantly decreased, compared with the HC group (78.7 [52.7–121.2] vs 292.4 [210.2–426.5] pg/mL, *P* <0.001; 95.8 [71.7–126.3] vs 292.4 [210.2–426.5] pg/mL, *P* <0.001). SCH group showed a higher FGF19 concentration than OH group, but there was no significant difference between the 2 group (78.7 [52.7–121.2] vs 95.8 [71.7–126.3], *P *>0.05). Additionally, we observed a significant higher level of FGF19 in isolated TPOAb group compared with OH and SCH groups (78.7 [52.7–121.2] vs 252.0 [205.9–353.5], *P* <0.001; 95.8 [71.7–126.3] vs 292.4 [210.2–426.5] pg/mL, *P* <0.001). However, the difference between Isolated TPOAb group and HC was not significant (252.0 [205.9–353.5] vs 292.4 [210.2 to 426.5] pg/mL, *P* >0.05).

**Table 1:**
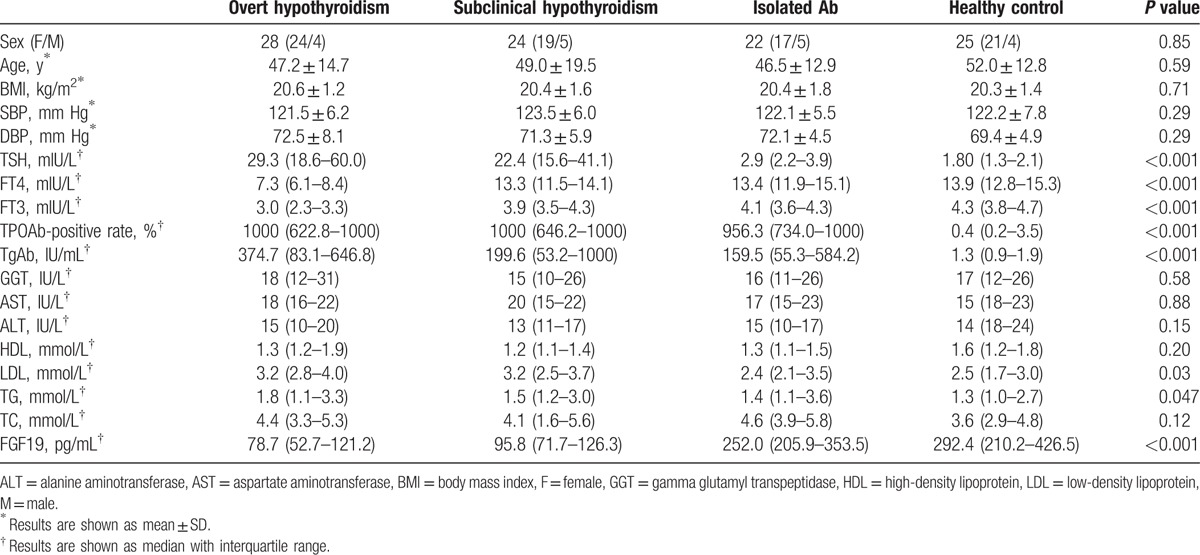
Characteristics of the study population.

### 3.2 TSH independently associated with Serum FGF19

**Figure 3:**
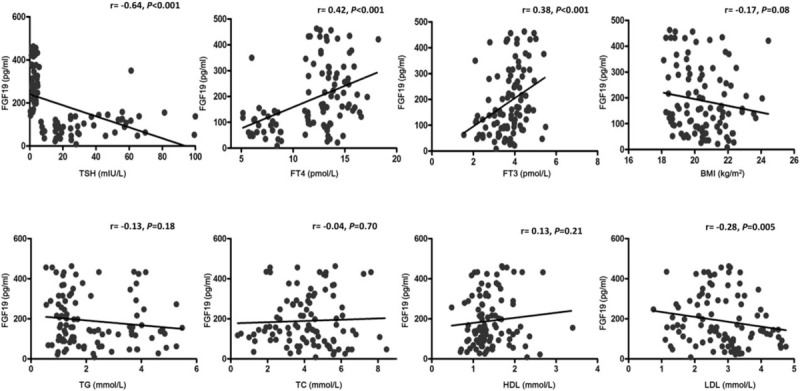
FGF19 was correlated positively with FT4 and FT3, but correlated negatively with TSH and LDL. LDL = low-density lipoprotein, TSH = thyroid-stimulating hormone.

As shown in Fig. 3, we performed a correlation analysis between anthropometric, biochemical variables, and FGF19. FGF19 levels were found to be positively correlated with FT4 (*r* = 0.42, *P* <0.001) and FT3 (*r* = 0.38, *P* <0.001), but negatively correlated with TSH (*r* = −0.64, *P* <0.001) and LDL (*r* = −0.28, *P* = 0.005). As shown in Table 2, to control the confounding factors, multiple linear regression analysis using FGF19 as the dependent variable identified Log TSH as an independent predictor after adjustments for Log FT4, Log FT3, Log LDL, Log HDL, and Log TC.

**Table 2:**
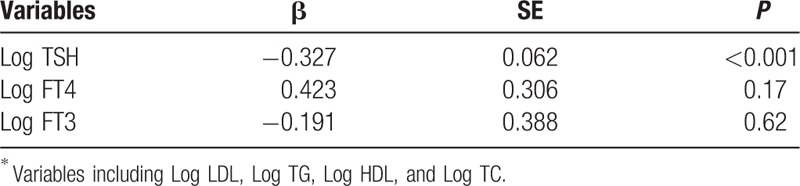
Variables independently associated with FGF19, as identified by linear regression analysis^∗^.

## 4 Discussion

To our knowledge, this is the first comprehensive study to evaluate the association between FGF19 and thyroid dysfunction. In this study, we have demonstrated for the first time that circulating FGF19 concentrations were significantly increased in patients with OH and SCH compared with the HC group and isolated TPOAb group. Serum FGF19 concentrations were independently associated with TSH concentrations.

Accumulating evidence suggests that multiple circulating cytokines are correlated with lipid and glucose metabolism.^[12,17,18]^ Furthermore, several independent groups have found that FGF19, as a newly emerging metabolic regulator, was highly likely associated with multiple metabolic disorders. Recently, Fang et al showed that FGF19 levels are decreased in subjects with impaired fasting glucose and inversely associated with fasting plasma glucose levels. Almost at the same time, Wang et al^[19]^ found that FGF19 was decreased in gestational diabetes patients. Furthermore, Alisi et al^[20]^ demonstrated FGF19 in patients with NAFLD. Hao et al^[21]^ showed serum levels of FGF19 were inversely associated with coronary artery disease. The effects of FGF19 on regulating energy homeostasis were first discovered in transgenic mice with overexpressed FGF19. Tomlinson's group observed an increased energy expenditure level, a significant decreased fat mass, as well as resistance to diet-induced obesity in the transgenic mice.^[3]^ Administration of recombinant FGF19 protein recapitulated most of these metabolic effects, preventing both high fat diet-fed and ob/ob mice from the impairment of glucose intolerance.^[4]^ Although the molecular mechanisms still remain unclear, they believe that FGF19 may play a role in the pathogenesis of metabolic disorders. Together, these findings make scholars believe FGF19 is a promising regulator in glucose and lipid metabolism.^[22]^

Hypothyroidism has been regarded as a great hazard to human health. Studies have shown that OH and SCH are closely related to a variety of metabolic disorders including obesity, hypertension, dyslipoproteinemia, insulin resistance/diabetes mellitus, and cardiovascular diseases.^[15,16,23,24]^ Many metabolic regulators and adipokines/hepatokines including leptin, adiponectin, resistin, and FGF21 were found to be altered with thyroid dysfunction.^[25]^

Since the association between FGF19 and hypothyroidism is still unclear, our study aims to provide more evidence for people to understand the potential role of FGF19 in hypothyroidism. In the present study, we have observed that circulating FGF19 levels were decreased in patients with OH and SCH compared with the HC group. There was no significant difference between FGF19 levels in the isolated TPOAb group and the HC group. Multivariate regression indicated serum TSH levels were independently associated with circulating FGF19 levels. Therefore, it seems that thyroid insufficiency but not thyroid autoimmunity may have an impact on serum FGF19 concentrations. Furthermore, inconsistent with the previous study, the present study did not find any association of FGF19 with blood lipid profile.^[19,21]^ Since the mechanism by which the plasma FGF19 concentration is decreased remains unclear, it may be untimely to draw the implications of increased serum FGF19 concentrations in hypothyroidism at this point. However, recent studies demonstrated that TSH, through its receptor, triggered hepatic sterol regulatory element-binding protein (SREBP)^[26]^ and further study revealed that the SREBP negatively regulated the transcription of FGF19 in human intestinal cells; this suggested that SREBP may act as an upper stream of FGF19 and negatively regulated the expression of FGF19.^[27]^ The lower concentration of FGF19 in patients with OH and SCH shows that the pathways of energy metabolism are intermingled at a more fundamental level. This gives us a new insight as to how FGF19 works and on which further research may focus: whether there exists a TSH-SREBP-FGF19 pathway to better understand the intestinal-endocrine physiology. Also, the possibility is raised that FGF19 may serve as a more comprehensive target in those patients whose metabolic disorder symptoms are not fully improved by thyroid hormone replacement alone.

Consequently, there are also some limitations to our study. First, it was a cross-sectional study where changes in plasma FGF19 concentrations and a causal relationship between thyroid function and FGF19 functional concentration could not be addressed. Moreover, in this study, we allotted patients into groups according to thyroid function before randomly selecting. This method may have produced selection bias. Meanwhile, our study is limited by a small sample size. Therefore, more large-scale population-based studies are still needed to confirm our findings. Additionally, in our study, for we can hardly recruit adequate subjects to investigate the changes of serum FGF19 concentrations in patients with hyperthyroidism, more research is now needed to better understand the relationship of FGF19 with thyroid.

In summary, the present study first demonstrated that the circulating FGF19 level was decreased in patients with OH and SCH. Additionally, serum TSH was independently associated with serum FGF19 concentration. As the roles of FGF19 are more and more important in the pathogenesis of many metabolic diseases, we proposed that the thyroid hormone level should be taken into account when the serum concentration is explained. Further studies are needed to elucidate the role of FGF19 in the development of hypothyroidism and its bridging function in the connection of thyroid dysfunctions and metabolic diseases.

## Acknowledgments

We thank the patients and staff at Institute of Endocrinology of the First Affiliated Hospital of China Medical University for their involvement in this study.
